# In Situ Ethanolamine ZnO Nanoparticle Passivation for Perovskite Interface Stability and Highly Efficient Solar Cells

**DOI:** 10.3390/nano12050823

**Published:** 2022-02-28

**Authors:** Humberto Emmanuel Sánchez-Godoy, K. M. Muhammed Salim, Rubén Rodríguez-Rojas, Isaac Zarazúa, Sofia Masi

**Affiliations:** 1 Centro Universitario de los Lagos, Universidad de Guadalajara, Lagos de Moreno 47460, Mexico; humberto.sgodoy@academicos.udg.mx (H.E.S.-G.); rubenro@culagos.udg.mx (R.R.-R.); 2 Institute of Advanced Materials (INAM), Universitat Jaume I (UJI), Avenida de Vicent Sos Baynat, 12071 Castellon de la Plana, Spain; kunnumma@uji.es

**Keywords:** electron transport layer, ZnO nanoparticles, interface, organic ligands, surface passivation, perovskite, solar cells, stability, efficiency, charge recombination

## Abstract

Zinc oxide (ZnO) has interesting optoelectronic properties, but suffers from chemical instability when in contact with perovskite interfaces; hence, the perovskite deposited on the top degrades promptly. Surface passivation strategies alleviate this instability issue; however, synthesis to passivate ZnO nanoparticles (NPs) in situ has received less attention. Here, a new synthesis at low temperatures with an ethanolamine post treatment has been developed. By using ZnO NPs prepared with ethanolamine and butanol (BuOH), (E-ZnO), the stability of the FA_0.9_Cs_0.1_PbI_3_ (FACsPI)–ZnO interface was achieved, with a photoconversion efficiency of >18%. Impedance spectroscopy demonstrates that the recombination at the interface was reduced in the system with E-ZnO/perovskite compared to common SnO_2_/perovskite and that the quality of the perovskite on the top is clearly due to the ZnO in situ passivation with ethanolamine. This work extends the use of E-ZnO as an n-type charge extraction layer and demonstrates its feasibility with methylammonium perovskite. Moreover, this study paves the way for other in situ passivation methods with different target molecules, along with new insights regarding the perovskite interface rearrangement when in contact with the modified electron transport layer (ETL).

## 1. Introduction

Solar cells are typically composed of three elements: an electron transport layer (ETL) which is responsible of collecting negative charge carriers; the photoactive element in which the light absorption occurs to photogenerate the electrons and holes; and a hole transport layer (HTL), which collects the positive carriers. Perovskite materials have been at the center of the latest research in the field of photovoltaics due to their low cost and ease of synthesis, in addition to their properties such as high absorption coefficients and long diffusion lengths. Notably, perovskite solar cells (PSCs) have reached a record efficiency, of 25.5% to date [1], when metal oxide layers are used as electron or n-type transporters. Among the ETL candidates in perovskite solar cells, organic conductive materials such as graphene, fullerenes, and their derivatives have been extensively investigated [2,3,4]. These organic materials possess some advantages, such as a relatively easy solution synthesis from which the devices have shown good photovoltaic performance; however, these devices still have the major disadvantage of poor stability when exposed to environmental conditions such as temperature, humidity, or light. In order to solve this problem, metallic compounds based on TiO_2_, ZnO, and SnO_2_ have been investigated, such as Zn_2_SO_4,_ BaSnO_3,_ SrTiO_4_ [5,6,7]. TiO_2_ still as one of the most commonly used ETLs in perovskite sensitized solar cells due to its good electrical properties [8,9], but has a large disadvantage due to the poor control of the thickness and roughness of the film in a typical mesoporous layer. As a consequence, short circuits can occur in the solar cell, decreasing its efficiency. On the other hand, as HTL, Spiro-OMeTAD (2,2′,7,7,7′-tetrakis(N,N′-di-p-methoxyphenlamine)-9,9-spirobifluorene), and PTAA (poly-triarylamine) are commonly used with dopants such as Li-TFSI (bis(trifluoromethane)sulfonimide lithium salt) to increase their charge mobility and conductivity; however, this can lead to device degradation [10,11] due to oxidation related to unwanted ion migration and a chemical interaction with the perovskite. For this reason, strategies based on sulfonated phenothiazine-based are investigated to create strong Pb-S bonds to stabilize the whole device [12,13].

However, the choice of materials for maximizing efficiency is not always suitable for achieving maximum stability. Apart from the known sensitivity of perovskite to atmospheric conditions, [14] the degradation mechanisms at the interface with the electron transporting layer (ETL) have been studied with particular interest as they vary according to the nature of both the perovskite and the oxide. For instance, it has been shown that SnO_2_ is significantly more stable to the UV light than titania, [3] thus making the cells more stable over time. However, perovskites deposited on SnO_2_ have poorer thermal stability [15]. The same problem occurs for ZnO, which has not yet reached the performance levels of the other two oxides and is mainly used in the fabrication of laser and light emitting diodes (LEDs). The benefits of ZnO compared to polycrystalline TiO_2_ and SnO_2_ are improved conductivity and the wide direct bandgap of 3.37 eV, [16] which is better aligned with the perovskite conduction band [17] and improves the operating voltages. Furthermore, ZnO is characterized by superior mobility and greater transmittance in the visible/IR regions of the spectrum; thus, the photon can move freely and is easily absorbed by photoactive elements [18]. In other words, the introduction of ETL with higher electron mobility could result in an increase in the charge transport and a suppression of the recombination processes, thereby improving the performance of the solar cells [19].

ZnO has already been used as thin films for UV–Vis lasers, gas sensors, LEDs [20,21,22], and in dye-sensitized solar cells [23,24]. However, when considering the common use of ZnO as an ETL in perovskite solar cells, there still some limitations. Most notably, two major drawbacks stand out: (1) poor perovskite chemical stability on ZnO substrates; and (2) the ZnO/perovskite large interfacial charge recombination due to defects at the wurtzite surface, the most stable phase at ambient temperature [25]. The ZnO–perovskite interface instability has been widely reported, with a variety of degradation mechanisms proposed [4,26,27].

The difference between amphoteric ZnO and other metal oxides is that they can react with both acid and basic species, while other oxides are usually basic and react only with acid to form their respective salts. In the proposed reaction with perovskite, it was first hypothesized that CH_3_NH_3_I (MAI) decomposed into CH_3_NH_2_ + HI, where the HI could react with the amphoteric ZnO surface to form ZnI_2_ and H_2_O. While the basic structural characterization was unable to detect ZnI_2_ [27], computational studies demonstrated the easy deprotonation of CH_3_NH_3_^+^ by ZnO, which was accelerated by hydroxyl groups or acetate ligands on ZnO NPs surface, and resulted not in the formation of ZnI_2_, but in the formation of zinc hydroxide (ZnOH); higher temperature annealing of ZnO decreased the surface density of hydroxyl groups, but was not enough to improve perovskite stability [4]. More recently, the degradation mechanisms at the MAPbI_3_ (MAPI)–ZnO interface were also experimentally demonstrated by detection of ZnOH with Fourier transform infrared (FTIR) spectroscopy of ZnOH [28]. Moreover, by replacing the MA^+^ (pKa ≈ 10.6) with the more acidic formamidinium (FA^+^, pKa ≈ 11.5), the solar cells showed enhanced UV stability (500 min) due to the positive charge resonance stabilized between the two “N” atoms, especially if a C_60_ layer was used to passivate the ZnO surface, and the thermal stability also approached that of the SnO_2_ control device (150 min) [28]. As the ZnO can react with both the cations, the reason why the FA^+^-based perovskite is more stable than the MA^+^-based material is in the kinetics of the reaction, which are predicted to be significantly slower compared to those for the MA^+^ as a result of the lower acidity of FA^+^.

Despite these steps forward, the planar devices based on ZnO ETL without passivation layers generally suffer from severe hysteresis due to the carrier accumulation at the interface of perovskite/ETL [29] To partially solve this problem, the use of ZnS has been exploited either to passivate the interface or to take benefits from the S atoms, which coordinate with Pb^2+^ ions, forming a pathway for electron transfer, thus accelerating charge extraction and reducing hysteresis. In line with this, a hysteresis-free solar cell with a PCE of 20.7% was achieved by interface sulfidation with ZnS [30], maintaining 88% of their initial performance for 1000 h under storage conditions and 87% for 500 h under UV radiation. Moreover, in this way, a PCE of 20% was achieved by introducing an interlayer, such as magnesium oxide, polyethanolamine, or polyethylenimine [31,32,33], between the ZnO and the perovskite film, but the stability was still not comparable with other metal oxide-based solar cells.

Additionally, it has been demonstrated that the ZnO synthesis method plays an important role in the performance of perovskite over time [34]. Several synthetic methods for ZnO NPs are reported [22]; most are characterized by high temperature, but the current goal is to synthesize these materials at lower temperatures than used for TiO_2_ or SnO_2_ processed in solution, making the methods suitable not only for large-scale processes with considerable energy savings, but also for deposition on flexible substrates [34].

Here, a simple ZnO synthesis method, at a temperature as low as 65 °C, was developed for use in perovskite solar cells without interlayers at the interface between ZnO and perovskite. The strategy comprised in situ passivation by post treatment of the synthesized ZnO surface with alternative basic ligands, such as ethanolamine and BuOH (E-ZnO). Since ZnO acts as a base in the reaction with the acidic perovskite, the in situ passivation with another basic species, such as the amine, contributes to the slowing of the reaction at the E-ZnO/perovskite interface. The positive effects are clear for FA_0.9_Cs_0.1_PbI_3_ (FACsPI), but promising results were obtained also for MAPI, demonstrating the universal advantage of the novel in situ passivation method. The results were improved photovoltaic performance, with an efficiency of ~18% and improved stability (for one week) in ambient conditions (25 °C, relative humidity (RH) of 50%) without encapsulation. Moreover, impedance measurements demonstrated that E-ZnO reduces the recombination resistance compared to common SnO_2_, due to the surface in situ passivation mechanism for the ethanolamine post treatment, in line with the improved stability achieved. A simulation study to correlate the experimental results and photogeneration process showed improved charge transport properties after the preparation of the devices, which was due to the better quality of the perovskite/ETL interface.

## 2. Results

**Figure 1:**
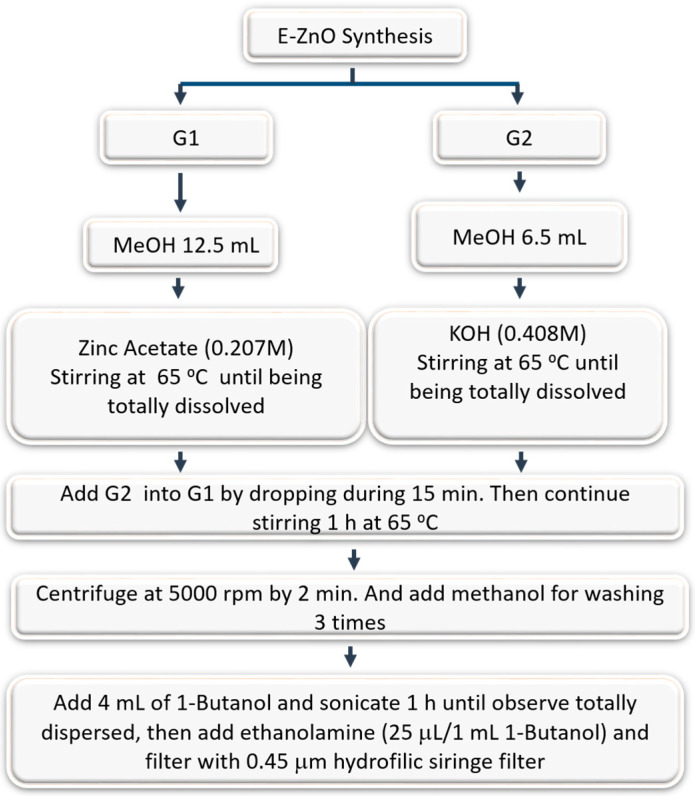
Schematic diagram of the synthesis of E-ZnO NPs in this work.

Generally, with the reported synthesis procedures, ZnO is obtained with a wurtzite crystalline structure [29]. This structure is characterized by some intrinsic defects, such as interstitial and oxygen vacancies, which act as charge trapping sites when it is used as an ETL. These defects provoke recombination of the photogenerated electrons and holes when the perovskite solar cell is illuminated [35,36,37,38]. Additionally, pristine ZnO presents a big number of OH groups on its surface, which dramatically reduces the conductivity of the material and promotes the degradation of the perovskite on the top [26]. In this work, to overcome these issues, the ZnO NPs surface was functionalized with butanol (BuOH) and ethanolamine (see details in the Experimental Section and Figure 1 and Figure S1). The Raman and Fourier-transform infrared (FTIR) spectra of the ZnO NPs with and without ethanolamine are shown in Figure S2a,b, in which the peaks of ethanolamine can be observed. In addition, Figure S2c shows the transmission electron microscopy (TEM)—energy-dispersive X-ray spectroscopy (EDS) spectra of ZnO and E-ZnO NPs, with the relative ratio of Zn and O different for each system. The higher ratio of oxygen in E-ZnO has been attributed to the surface ethanolamine passivating ligands. Moreover, the peaks at 31.6° (100), 34.4° (002), 36.2° (101), and 47.5° (102) in the XRD pattern are ascribed to the crystalline diffraction of ZnO NPs (Figure S2d). This strategy allows passivation of the surface with basic species, which have the second advantage of being less reactive in contact with the perovskite, and thereby avoiding side reactions that lead to premature degradation, as illustrated below.

**Figure 2:**
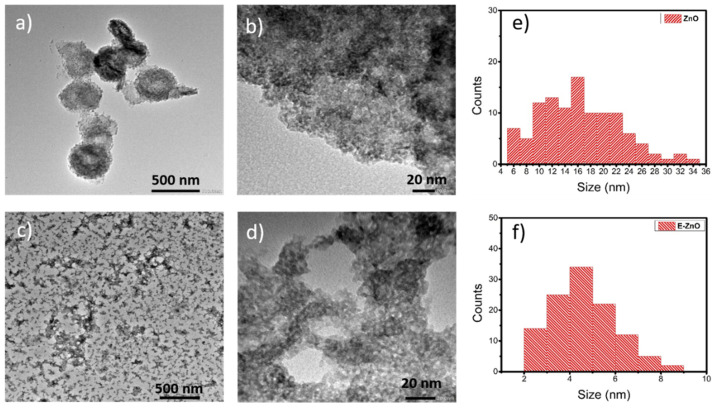
Representative TEM micrographs of (**a**,**b**) ZnO NPs and (**c**,**d**) E-ZnO NPs, and corresponding histograms of the size distribution of ZnO (**e**) and E-ZnO (**f**).

TEM micrographs of ZnO NPs and ethanolamine-functionalized ZnO NPs (E-ZnO) are shown in Figure 2a–d. ZnO NPs present a wide distribution of particle size (from 5 nm to 35 nm); meanwhile, Figure 2e,f show E-ZnO NPs with a homogeneous size distribution of between 2 and 9 nm. Moreover, the ZnO NPs in Figure 2a are agglomerated while the E-ZnO NPs in Figure 2c are well dispersed, due to the functionalization, which allows for a better dispersion in the solvent used.

In order to replace SnO_2_ with ZnO, a comparative analysis was carried out. In the optical characterization (Figure S3), both ZnO and SnO_2_ compact layers show about 90% of transmittance for λ > 450 nm. However, for lower wavelengths, ZnO has considerably lower transmittance than SnO_2_, reaching 72% and 87% at 320 nm, respectively. The reduction of the ZnO transmittance in the UV region could be attributed to its bandgap, which is around 3.2 eV (nearly 0.8 eV lower than in SnO_2_).

**Figure 3:**
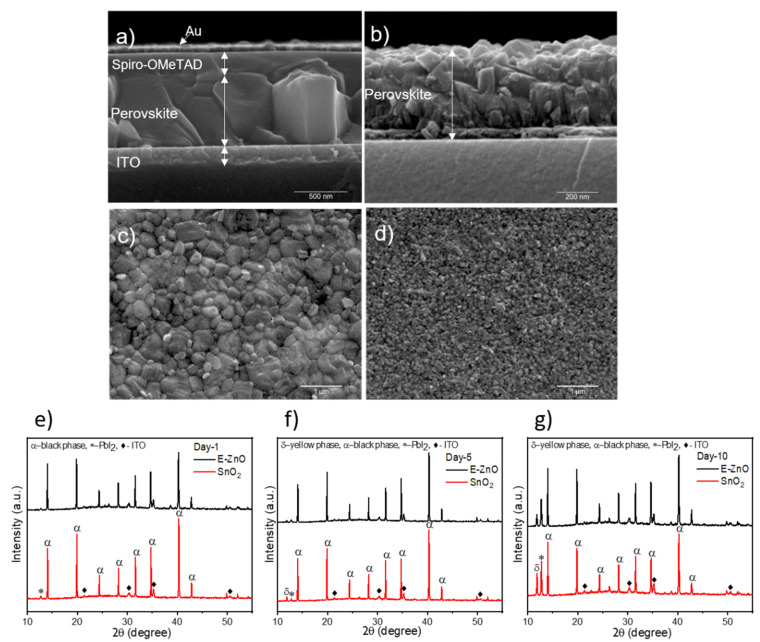
Representative SEM images of (**a**) SnO_2_-based FACsPI solar cell cross-section, (**b**) E-ZnO-based FACsPI solar cell cross-section. Top view of (**c**) FACsPI on the top of SnO_2_ and (**d**) FACsPI on the top of E-ZnO. Perovskite on the top of ZnO was not measured owing to lack of stability. X-ray diffraction patterns of the perovskite (FACsPI) over SnO_2_ and E-ZnO after (**e**) 1 day, (**f**) 5 days, and (**g**) 10 days.

After the perovskite deposition on the top of ZnO, both MAPbI_3_ and FACsPI perovskites degrade in a few minutes; thus, we discarded the ZnO prepared by standard methods and focused on E-ZnO, using samples on SnO_2_ as reference. First, the FACsPI was deposited on the top to observe the effects on perovskite formation. Scanning electron microscopy (SEM) analysis was carried out using SnO_2_ as reference ETL. The perovskite grains grown on SnO_2_ (Figure 3 and Figure S4a) have a diameter of 325 ± 125 nm, and form a very homogeneous layer with a thickness of 630 nm, where each grain has the thickness of the film (Figure 3a), like previous reports [39] of flat perovskite devices with SnO_2_ as the ETL. On the other hand, perovskite grains grown on E-ZnO (Figure 3d and Figure S4b), were considerably smaller (112 ± 36 nm and 90 nm of height) and the resulting perovskite films were almost half of the thickness (368 nm, see Figure 3b) than those obtained on SnO_2_.

The X-ray diffraction (XRD) patterns of FACsPI perovskite films show the expected stable phase (α-phase) in both types of ETL (Figure 3e–g). After 5 days of storage under ambient conditions (T = 20 °C; relative humidity: 40%), the XRD pattern of the perovskite on SnO_2_ showed a small peak at 12.7° attributed to PbI_2_, which indicates perovskite degradation; for perovskite on E-ZnO, the peak is almost imperceptible (Figure 3f). After 10 days, the PbI_2_ peak and the peak at 11.8° of the δ-phase are more prominent in perovskite with SnO_2_ as the ETL compared to one of the perovskites deposited on the top of the E-ZnO (Figure 3g). Thus, the functionalized ZnO used in this work allows for perovskite deposition on the top of ZnO and achieves greater stability compared to standard ETL.

As the improved stability of the ZnO–perovskite interface by adding the new amine functionalization has been corroborated, the device performances have been investigated. Comparison with common ZnO-based devices will be not reported owing to the inability to measure reliable samples and devices due to the fast degradation process.

**Figure 4:**
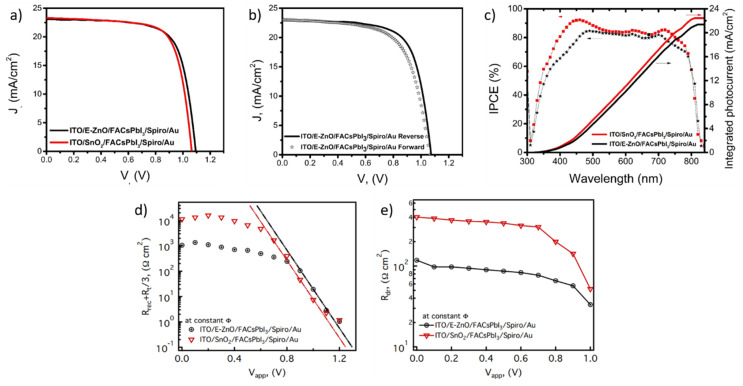
Photovoltaic parameters from optimized FACsPI solar cells with SnO_2_ and E-ZnO as ETL: (**a**) J/V curves, (**b**) forward and reverse bias with minimal hysteresis, (**c**) incident photon-to-current efficiency curves and integrated photocurrent, (**d**) resistances associated with transport and recombination processes R_t_/3 +R_rec_, and (**e**) R_dr_ obtained from the impedance characterization and the model presented in Figure S5.

The photovoltaic performances of flat devices using SnO_2_ and E-ZnO as the ETL have been investigated (Figure 4a and Table 1). It has been found that both kinds of optimized samples have a very similar photoconversion efficiency (PCE = 17.9 and 18.1% for SnO_2_ and E-ZnO based samples, respectively), with the main difference being that E-ZnO based samples have a slightly lower current density (J_sc_) compared with SnO_2_ samples (23.07 mA/cm^2^ and 23.30 mA/cm^2^, respectively) and, especially, a higher open-circuit voltage (V_oc_) (1.09 V and 1.07 V). The reduction of J_sc_ is in line with the reduction of the optical density (Figure S3c) of the film due to its lower thickness. On the other hand, the increase in the V_oc_ could indicate a reduction in the recombination processes. Moreover, the device based on E-ZnO is free of hysteresis (Figure 4b), thus demonstrating that the in situ passivation method adopted in this study has the dual advantage of improving the perovskite stability while reducing the solar cell hysteresis, if compared to the interlayers previously reported in literature, as commented above.

To shine a light on the small but significant variations of J_sc_, incident photon-to-current efficiency (IPCE) analyses have been carried out; representative results of both kinds of samples are plotted in Figure 4c. It can be observed that SnO_2_-based perovskite solar cells have an IPCE over 80% in the range 380–800 nm, with a maximum of 90% at 450 nm and a small peak that surpasses 85% at 700 nm; these characteristics are consistent with previous reports of this type of solar cells [39]. When SnO_2_ is replaced by E-ZnO, a general reduction in the IPCE is obtained, reaching a maximum value of 80% that is almost constant in the 450–700 nm range and a quite strong reduction of efficiency (IPCE = 60%) in the 380–450 nm range. The small IPCE differences in the range 450–700 nm are attributed to a lower photogeneration process due to the thinner perovskite films obtained when E-ZnO is used as the ETL (Figure 3a,b). Furthermore, the strong IPCE differences in the 380–450 nm range are more consistent with a reduction in the incoming photons at the active layer due to the lower transmittance of ZnO in the UV region (Figure S3b).

Moreover, by impedance measurements at fixed irradiance and scanning upon the applied voltage, the internal charge transport, and recombination processes are characterized in order to compare the E-ZnO and the SnO_2_. Figure 4d,e shows the resistance commonly observed in solar cells, including dye-sensitized, quantum dots sensitized, and perovskite solar cells, and indicates the presence of two parallel processes where the one with the lower resistance becomes dominant [40,41]. Recombination resistance (R_rec_) and transport resistance (R_t_) exponentially decrease with voltage; then, the exponential trend observed at V_app_ > 0.8 V could be attributed to the addition of the recombination resistance plus one-third of the transport resistance, as proposed by Yoo et al. [42] In contrast, even if its physical nature needs more investigation, the resistance trend at voltages of <0.8 V could be related to leakage processes [41].

By analyzing the exponential region of the curves in Figure 4d, it is observed that the samples made with E-ZnO have higher resistance than those made with SnO_2_. This increase in resistance could be originated by two processes: (1) an increase in the transport resistance, which would be manifested as a reduction in the fill factor; and (2) an increase in the recombination resistance, which would produce an increase in the V_oc_ [43]. According to the photovoltaic characterization (Figure 4a and Table S1), when E-ZnO is used as the ETL, a very small reduction in the FF is obtained (0.3% relative decrement). Meanwhile, the V_oc_ increment is significant (1.9% relative enhancement). Therefore, the observed resistance enhancement in the high-voltage region indicates that the introduction of E-ZnO as the ETL reduces the recombination processes due to in situ passivation of the ethanolamine on the E-ZnO surface. The Shockley–Read–Hall theory [44] indicates two ways to reduce superficial recombination [45]. One is by decreasing the density of superficial states, which can be achieved by obtaining a smaller surface area. This is attained by increasing the grain size, which does not occur in the synthesis proposed in this work, as mentioned in the morphological analysis (Figure 2e,f). The other way to reduce superficial recombination is by decreasing the free carrier’s concentration through surface passivation, normally achieved by doping [38]. In this work, the surface in situ passivation was carried out by the replacement of the hydroxyl groups by ethanolamine, also causing a uncharged surface due to the ethanolamine neutral ligands [32], which triggered the desired contact between E-ZnO and perovskite.

Figure 4e shows the discharge-like resistance (R_dr_) behavior as a function of the applied voltage; here, it can be observed that both kinds of samples have an almost constant value of R_dr_ in a wide range of voltages, but decrease when V_app_ > 0.7 V. It is very important to note that R_dr_ is almost four times smaller in the samples made with E-ZnO as ETL. It has been hypothesized that this resistance could be associated with the ionic mobility in the perovskite [43], then the R_dr_ reduction when E-ZnO is used could imply that ions have more mobility in this kind of sample, probably due to the smaller grain size of these samples (see Figure 3d), which imply more grain boundaries where the ions could easily move [46].

Although the samples with E-ZnO as the ETL have similar performances to the those using SnO_2_, the question is whether the presence of ethanolamine removes free radicals on the E-ZnO surface, such as the OH groups on common ZnO, which could promote faster degradation processes, thereby reducing the stability of the device under ambient conditions (25 °C and 35% of relative humidity).

To evaluate the effect of our passivated E-ZnO NPs on the stability of the devices, the photovoltaic performance of the solar cells was measured after 2 and 6 days of their fabrication. Figure S6 and Table S2 show the evolution of the different photovoltaic parameters over 6 days in representative samples made with E-ZnO and SnO_2_ as the ETL. Furthermore, statistical plots and representative J/V curves are reported in Figure S7a–d. Figure S7 demonstrates that the use of E-ZnO not only gives better stability (see the image in Figure S8) than SnO_2_, but even increases the average PCE with time. In detail, E-ZnO-based perovskite solar cells show a relatively good initial photovoltaic performance with a maximum PCE of 14% (average PCE = 10.84%, V_oc_ = 1.01 V, J_sc_ = 17.04 mA/cm^2^, and FF = 62%) and then a constant increase of the photovoltaic parameters after 2 days with a maximum PCE of 16.1% (average PCE = 14.55%, V_oc_ = 1.06 V, J_sc_= 20.48 mA/cm^2^, and FF = 66.76%). After 6 days, the maximum PCE of 17.35% and averages of PCE = 15.94%, V_oc_ = 1.06 V, J_sc_ = 22.09 mA/cm^2^, and FF = 68.09% were measured (Figure S7 and Table S2); meanwhile, the reference cells (SnO_2_-based perovskite solar cells) showed a slight decrease until reaching stable values of 11.26% of PCE (V_oc_ = 1 V, J_sc_ = 22 mA/cm^2^, FF = 52.54%, max PCE = 14.24%). This improvement in the performance of E-ZnO samples is principally due to a notorious J_sc_ enhancement and small increases of FF and V_oc_. These results suggest that in situ passivation treatment of the E-ZnO NPs effectively reduces the destructive interactions of the ZnO free radicals with the perovskite crystals, increasing their stability (Figure S8) as shown in XRD patterns (Figure 3e–g) and making the E-ZnO NPs a very promising material for application in perovskite solar cells.

In addition, polymer interlayers, such as polyvinylpyrrolidone (PVP) and polymethylmethacrylate (PMMA), were used at the ZnO–perovskite interface (Figures S9 and S10) to compare the efficiency of our method without a passivation layer, known to be used as a standard strategy in the fabrication of ZnO-based solar cells. In addition, in these conditions, a slight increase in the photovoltaic performance over time is observed, but they were not as efficient as bare E-ZnO-based perovskite solar cells.

In order to prove the robustness of the method, we used E-ZnO with the more sensitive MAPI as an active layer. The MAPI perovskite was not stable on the top of ZnO when synthesized by the reported methods described in the experimental sections (Figure S11b); however, with E-ZnO, the PCE of the solar cells improved threefold (from 2.9% to 8%) (Table S3).

**Figure 5:**
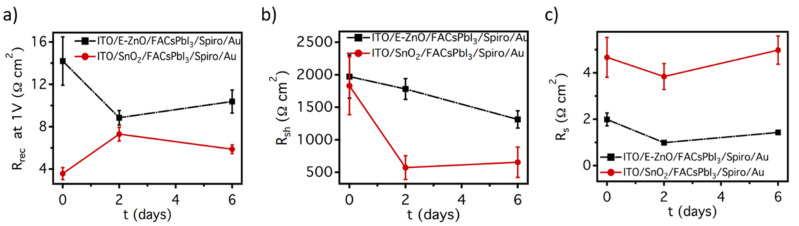
Temporal evolution of the (**a**) recombination, (**b**) shunt, and (**c**) series resistances, obtained from the simulation of the J/V curves measured for solar cells at 0, 2, and 6 days since their fabrication.

A simulation study to understand the improvement in current over time has been exploited to find a correlation between the experimental results and the photogeneration processes. In general, the solar cell performance is tuned by the combination of several processes. J_sc_ is determined by the product of the photogeneration and the charge collection efficiency (AKA internal quantum efficiency, IQE) at the time, determined by the equilibrium of the charge transport and recombination processes. The V_oc_ is determined by the equilibrium of the photogeneration and the charge recombination and leakage processes. Finally, the FF is molded by the combination of the charge recombination, leakage, and transport processes [43]. To better understand how these internal processes were modified over time and their correlation with the observed changes in the solar cell performances, the J/V curves were fitted using a simple diode model [41] (detailed description is provided in equations 1 to 5 of the Supporting Information), where the recombination resistance (R_rec_), the shunt resistance (R_sh_), and the series resistance (R_s_) represent the opposition to the recombination, leakage, and transport processes, respectively. The obtained fittings have a high degree of adjustment, as shown in the representative examples in Figure S12, obtaining a correlation factor over 0.998 in all samples. Fitted R_s_, R_sh,_ and R_rec_ at 1 V as a function of time are shown in Figure 5. Here, it is observed that the E-ZnO samples always have higher R_rec_ and R_sh_ values than SnO_2_ samples. This implies that when similar photogeneration currents are obtained (and hence similar J_sc_ values), E-ZnO samples will have a higher V_oc_, as observed in Figure S12. On the other hand, E-ZnO samples always have smaller R_s_ values than the SnO_2_ samples. The combination of higher R_sh_ and smaller R_s_ is principally responsible for the better FF performance of the E-ZnO samples (Figure 4). Through the detailed analysis, it was observed that R_rec_ in E-ZnO samples was reduced to nearly two-thirds of the original value in the first 2 days, remaining relatively stable even with a small increase until day 6. These results suggest that in the first burn-in stage, samples based on the perovskite layer on the E-ZnO start to form new recombination sites, probably due to ion migration at the grain boundaries. However, the formation of new recombination sites stops at day 2, maintaining a lower recombination rate than in SnO_2_ samples, probably due to the in situ passivation effect of the ethanolamine on the E-ZnO surface [32]. On the other hand, it is observed that R_sh_ linearly decreases with time in E-ZnO samples, from 2000 to 1500 Ω, (Figure 5b). Meanwhile, in SnO_2_ samples, R_sh_ exponentially decreases until reaching 500 Ω. This parameter is related to the shortcuts in the sample due to the presence of pinholes and irregularities in the perovskite films usually formed during the dissolution and recrystallization processes typically observed during perovskite degradation [47]. The observed behaviors indicate that E-ZnO film reduces the formation rate of this kind of perovskite imperfections. This effect could be attributed to a reduction in the residual PbI_2_ at the ETL perovskite interface due to electrostatic interactions between ethanolamine and I^−^ [32]. Finally, both kinds of samples have similar R_s_ trends, with a small resistance decrease in the first 2 days followed by an increase in similar magnitude (Figure 5c). This indicates that the charge transport properties are improved in the first 2 days, probably by improvement of contact at the perovskite/ETL interface due to the ion rearrangement. Then, after 6 days, the devices stabilized, reaching an Rs similar to the fresh devices.

## 3. Conclusions

In summary, we have achieved stable perovskite on the top of the ZnO layer with simultaneous high-efficiency and hysteresis-free solar cells based on ZnO electron transporting material passivated with ethanolamine (E-ZnO). The strong interactions between ZnO NPs and perovskite surface are avoided by the addition of basic ethanolamine in situ after the synthesis. Simultaneously, E-ZnO improved the charge transport properties of the resulting devices based on FA_0.9_Cs_0.1_PbI_3_. The resulting solar cells demonstrated a PCE close to the state of the art, with a high PCE of 18.1%, while the perovskite films were stable for 10 days. In addition, the use of E-ZnO in perovskite solar cells promoted an improvement in open-circuit voltage and fill factor, due to better charge transport, while maintaining similar current values in comparison to the reference cells based on SnO_2_. More importantly, due to benefits from ethanolamine in situ passivation, the stability of the more sensitive perovskite MAPbI_3_ is demonstrated, with devices exhibiting a threefold increase in PCE, to over 8%, presenting an important breakthrough in solar cells based on ZnO NPs.

Impedance spectroscopy also demonstrated that the recombination at the interface is reduced in the E-ZnO/perovskite system compared to the common SnO_2_/perovskite, highlighting the benefits of ZnO as the electron transporting material. We foresee that the in situ passivation strategy in the present work would also be insightful for rationally modulating the surface chemistry of various types of transporting layers for their applications in different optoelectronic devices, e.g., LEDs and detectors, thereby achieving improved performance.

## Figures and Tables

**Table 1:** Photovoltaic parameters of FACsPI and MAPI solar cells with SnO_2_, ZnO, and E-ZnO as ETL.

	PCE (%) (Mean ± SD)	VOC (V) (Mean ± SD)	JSC (mA/cm^2^) (Mean ± SD)	FF (%) (Mean ± SD)	BEST PCE (%)
**SnO_2_ FACsPI**	17.06 ± 0.77	1.05 ± 0.01	23.15 ± 0.25	70.1 ± 2.49	17.90
**ZnO FACsPI**	/	/	/	/	/
**E-ZnO FACsPI**	17.16 ± 0.44	1.08 ± 0.01	22.90 ± 0.25	69.5 ± 1.09	18.10
**SnO_2_ MAPI**	13.54 ± 1.06	1.01 ± 0.04	18.68 ± 0.69	71.43 ± 5.40	15.70
**ZnO MAPI**	1.67 ± 1.00	0.76 ± 0.04	4.15 ± 0.73	45.53 ± 12	2.90
**E-ZnO MAPI**	6.23 ± 1.30	0.98 ± 0.005	12.09 ± 1.3	52.35 ± 7.30	8.03

## Supplementary Materials

The following supporting information can be downloaded at: https://www.mdpi.com/article/10.3390/nano12050823/s1, Figure S1: Schematic diagrams of the ZnO NPs synthesis for solar cells reported in the literature; in (a) for ZnO based quantum dots solar cells^13^ (synthesis 1) and (b) ZnO based perovskite solar cells ^18^ (synthesis 2), Figure S2: (a) Raman and (b) FTIR spectra (C-N bonds at 1,000 cm^−1^, C-H at 3,000 cm^−1^ and OH at 3,500 cm^−1^); (c) TEM-EDS spectra of ZnO and E-ZnO NPs, the ratio of Zn and O is different for each system. The higher ratio of oxygen in E-ZnO has been attributed to the ethanolamine passivating ligands; (d) XRD pattern of ZnO powder on ITO surface., Figure S3: UV-Vis (a) Absorption and (b) transmittance spectra of the E-ZnO and SnO_2_ compact layers deposited on the ITO. C) Optical absorbance of FACsPI on SnO_2_ and E-ZnO, Figure S4: Size distribution of the perovskite grains deposited over (a) SnO_2_ and (b) E-ZnO, mean size ± standard deviation is reported, Figure S5: The equivalent circuit used to fit the impedance response of the samples, showing the series resistance R_s_, the resistance associated with Transport and recombination processes R_t_/3 +R_rec_, the geometrical capacitance C_g_, and the relaxation like resistance and capacitance R_dr_ and C_dr_^32^, Figure S6: Temporal evolution of photovoltaic parameters the devices using E-ZnO and SnO_2_ as ETL; (a) PCE, (b) V_oc_, (c) J_sc_ and (d) FF, Figure S7: Statistical analysis of photovoltaic parameters of (a) day 0, (b) day 2, (c) day 6 and (d) representative J/V curves of the performance evolution of SnO_2_ and E-ZnO based FACsPI solar cells, Figure S8: Picture of FACsPI perovskite over SnO_2_ and E-ZnO after 2 days in ambient conditions, no degradation is observed, Figure S9: Statistics of photovoltaic parameters of SnO_2_, E-ZnO, ZnO/PVP and ZnO/PMMA based FACsPI solar cells at (a) day 0, (b) day 2 and (c) day 6, Figure S10: Temporal evolution of (a) PCE, (b) V_oc_, (c) J_sc,_ and (d) FF of FACsPI solar cells with different ETL: SnO_2_, E-ZnO, ZnO/PVP, and ZnO/PMMA, Figure S11: Best J/V curves of ZnO-based MAPbI_3_ solar cells made with (a) synthesis 1; (c) synthesis 2 and (d) E-ZnO, and (b) picture of degraded MAPbI_3_ perovskite over ZnO layer, Figure S12: (a) Representative fittings of the J/V curves using the diode model compared with the experimental data. All simulated curves had a correlation factor higher than 0.995. (b) Representative simulations and experimental data of the resistances in the J/V curves title, Table S1: Photovoltaic parameters of optimized SnO_2_ and E-ZnO based FACsPI perovskite solar cells, Table S2: Photovoltaic parameters of SnO_2_ and E-ZnO based FACsPI perovskite solar cells during 6 days, Table S3: Statistic of the MAPbI_3_ solar cells figures of merits, based on ZnO of synthesis 1, synthesis 2, and E-ZnO.
