# Supplementary material for: In Situ Ethanolamine ZnO Nanoparticle Passivation for Perovskite Interface Stability and Highly Efficient Solar Cells
Source: Nanomaterials (Basel). 2022 Feb 28;12(5):823. doi: 10.3390/nano12050823 (PMC8912770; doi:10.3390/nano12050823)
Supplement: Supplementary file 1 [file nanomaterials-12-00823-s001.zip › nanomaterials-1583109-supplementary.pdf]

Supplementary Information

In Situ Ethanolamine ZnO Nanoparticle Passivation for Perovskite Interface Stability and Highly Efficient Solar Cells

Humberto Emmanuel Sánchez-Godoy [1,†], K. M. Muhammed Salim [2,†], Rubén Rodríguez-Rojas [1], Isaac Zarazúa [1,*] and Sofia Masi [2,*]

[1] Centro Universitario de los Lagos, Universidad de Guadalajara, Lagos de Moreno 47460, Mexico; humberto.sgodoy@academicos.udg.mx (H.E.S.-G.); rubenro@culagos.udg.mx (R.R.-R.)

[2] Institute of Advanced Materials (INAM), Universitat Jaume I (UJI), Avenida de Vicent Sos Baynat, 12071 Castellon de la Plana, Spain; kunnumma@uji.es

* Correspondence: isaac.zarazua@academicos.udg.mx (I.Z.); masi@uji.es (S.M.)

† These authors contributed equally.

Experimental Section

All the materials used for the experiments are reagent grade and were used as received. 2-propanol (99.7% from Carlo Erba), ethanol (96%) and acetone (99.25%) from PanReac, dimethylformamide (DMF), dimethyl sulfoxide (DMSO 99.9%), hydrochloric acid (HCl 37%), metallic zinc powder (99.995%), ethyl acetate, chlorobenzene (anhydrous 99.8%), acetonitrile (anhydrous 99.8%), 4-tert-butyl pyridine (96%), lithium bis (trifluormethylsulfonyl)imide (99.95%) were purchased from Sigma Aldrich while Spiro-OMeTAD (99%) from Feiming chemical limited, toluene (anhydrous 99.8%) from VWR and SnO_2 (15% in H_2O colloidal dispersion) precursor was obtained from Alfa Aesar, Indium Tin Oxide (ITO) coated glass substrates, lead iodide (98%) from TCI, formamidinium iodide (98%), cesium iodide (99%) and methylammonium iodide (99%)

Synthesis of ZnO and E-ZnO NPs: ZnO NPs were prepared by hydrolysis and the schematic diagram of the process is shown in Figure S1. In the beaker 1 (G1), 12.5 mL of MeOH and 0.295 gr of $(CH_3O_2)_2Zn$ were mixed on a stirrer hot plate at 65°C until the whole dissolution of the Zn precursor. At the same time in another stirrer hot plate is the beaker 2 (G2), 0.148 gr of KOH is dissolved in 6.5 mL of MeOH at 65 °C. Then, G2 was poured into the G1 by dropping for 15 min at 65°C to obtain ZnO NPs (NPs). ZnO NPs were washed 3 times by centrifuging at 5,000 rpm for 2 minutes and cleared with MeOH.[18] Finally to promote the ZnO surface functionalization, the washed NPs were re-dispersed in 4 mL of 1-Butanol and the solution sonicated for 1 hour; then ethanolamine was added in a ratio of (ethanolamine: ZnO solution = 25 L: 1 mL) and the resultant solution was filtered with 0.45 m hydrophilic syringe filter (Figure S1).

Solar cell fabrication: ITO substrates were etched with a mixture of metallic zinc powder and 6 molar HCl, then the substrates were brushed and sonicated with decon soap solution, deionized water, acetone, and isopropanol (15 minutes for each solvent). The substrates were dried with the air gun. Then, the substrates were treated with UV-ozone for 20 minutes;[38] for the SnO_2-based devices, 110 µL of 2.6% SnO_2 Alfa Aesar diluted solution were spin-coated for 40 sec at

3,000 rpm with a 3,000 rpm/sec acceleration ramp. For the ZnO-based devices, the same deposition parameters were used with the synthesized ZnO solution. Afterward, the substrates were cleaned around 5 mm to improve the contact with the gold with a wet cotton swap and annealed for 30 min at 150°C on a hot plate. When the substrates were cooled down they were treated with UV-Ozone for 60 minutes. At the same time a 1.4 molar $FA_{0.9}Cs_{0.1}PbI_3$ solution was prepared by sequentially dissolving in a 4:1 DMF:DMSO mixture at 70 °C Cs, FAI, and PbI_2, waiting to the complete dissolution of each powder before adding the next reagent. In the case of methylammonium perovskite, a 1.3 molar $MAPbI_3$ solution was prepared by sequentially dissolving in DMSO at 70°C MAI and PbI_2 waiting to the complete dissolution of each powder before adding the next reagent. After that under nitrogen atmosphere, 60 µL of perovskite was spin-coated according to the following steps: 10 sec to reach 2,000 rpm, 2 sec to 6,000 rpm, 28 sec at 6,000 rpm and stop in 1 sec, 100 µL of chlorobenzene was dropped as antisolvent at 25th sec after the beginning of the spinning. Then Perovskite layer was annealed for 10 min at 150°C. For the hole transport layer, 80 L of fresh Spiro-OMeTAD solution was spin-coated at 4,000 rpm for 30 sec over the annealed perovskite layer (it was prepared by 2 mL of chlorobenzene with 57.6 L of 4-tert-butyl pyridine and 35 L of Li-TFSI solution (520 mg of lithium salt in 1ml acetonitrile)). Finally, the 80 nm gold metallic contact layer was thermally evaporated.

Structural characterization: the morphologies of ZnO layers were carried out with transmission electron microscopy (TEM) W JEOL JEM 1010 using an accelerating voltage of 100 kV, with a resolution of 0.4 nm. The morphologies of the samples (ITO/SnO_2/FACsPI and ITO/E-ZnO/FACsPI) are carried out with a field emission scanning electron microscope (FEG-SEM) JEOL 3100F) operated at 5 kV.

Raman spectroscopy: the micro-Raman spectra were measured by WiTec apyron equipment at 532 nm excitation wavelength with an EMCCD detector. The laser power intensity used was 0.1 mW.

The FTIR spectra of the ZnO NPs are collected with a FTIR Equinox 55 (Bruker) with an ATR Pro (Jasco) equipped with a diamond crystal, in standard conditions (in the range 600-4,000 cm-1).

X-ray diffraction (XRD): The crystallographic information of the films were analyzed by X-ray diffractometer (D8 Advance, Bruker AXS) (Cu Kα, the wavelength of l = 1.5406 Å) within the range of 10–55° with a step size of 0.02°.

Incident-photon-to current conversion efficiency (IPCE): Measured using a QEPVSI-b Oriel measurement system and measured in DC mode.

UV-Vis Spectra: The absorptions spectra were obtained using a UV/Vis absorption spectrophotometer (Varian, Cary 300).

Solar cell characterization: the current–voltage (J/V) curves are measured using a Keithley 2612 source meter under AM 1.5 G (100 mW/cm^{-2}) provided by a Solar Simulator Abet with Xenon short-arc lamp Ushio 150 watts, in the air at a temperature around 25 °C and a relative humidity

around 40-50%, without encapsulation. Each curve was generated using 123 data points from a starting potential of 1.2 V to a final potential of -0.05 V (reverse scan; vice versa for the forward scan) using a scan rate of 10 mVs^{-1}. The measured active area of all cells was 0.121 cm^2 with 5 pixels each device, determined with a metallic external mask (a microscope is used to define the real active area). Photovoltaic performance of the aged devices was measured in ambient conditions (25 °C and RH 50%), after storing outside the glove box, without encapsulation, at ambient temperature, and at 1 sun.

The steady-state absorption spectra of the perovskite films were achieved by using a UV/Vis absorption spectrophotometer (Varian, Cary 300).

Electrochemical Impedance Measurements (EIS) were done applying a voltage perturbation of 20mV at different frequencies from 1MHz to 0.1Hz in a PGSTAT-30 from Autolab and under fixed irradiance of 1000 W/m^2 controlled by neutral density filters. The resulting spectra were fitted using the model proposed by Yoo et al. (see Figure S5)[32] obtaining the values of the circuit components with an error lower than 10%.

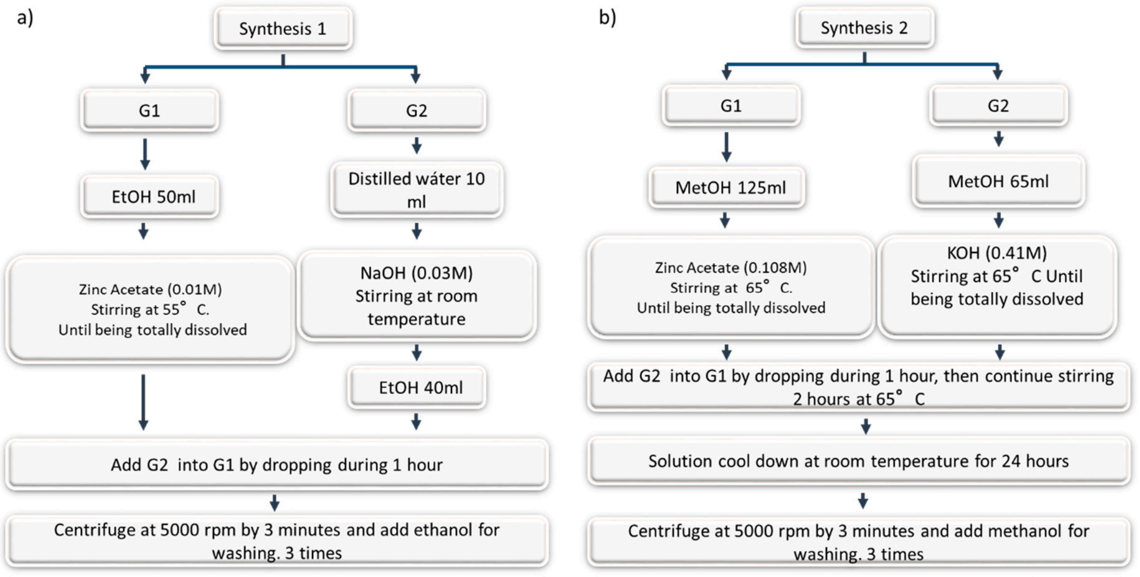

Figure S1. Schematic diagrams of the ZnO NPs synthesis for solar cells reported in the literature; in a) for ZnO based quantum dots solar cells[13] (synthesis 1) and b) ZnO based perovskite solar cells [18] (synthesis 2).

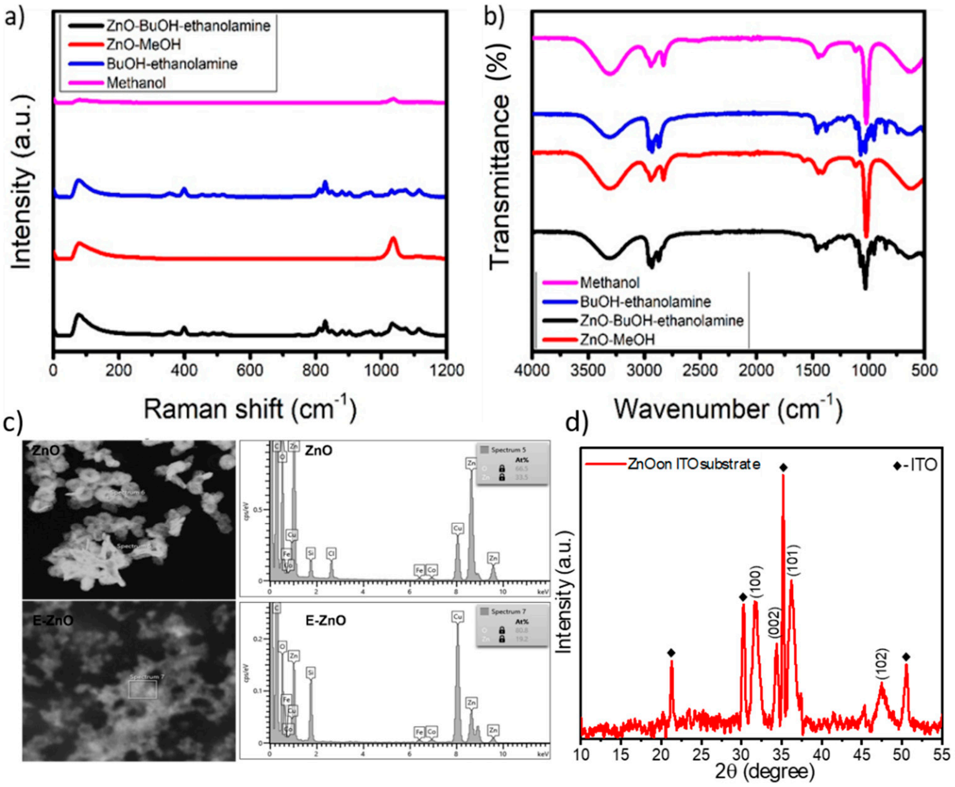

Figure S2. a) Raman and b) FTIR spectra (C-N bonds at 1,000 cm^{-1}, C-H at 3,000 cm^{-1} and OH at 3,500 cm^{-1}); c) TEM-EDS spectra of ZnO and E-ZnO NPs, the ratio of Zn and O is different for each system. The higher ratio of oxygen in E-ZnO has been attributed to the ethanolamine passivating ligands; d) XRD pattern of ZnO powder on ITO surface.

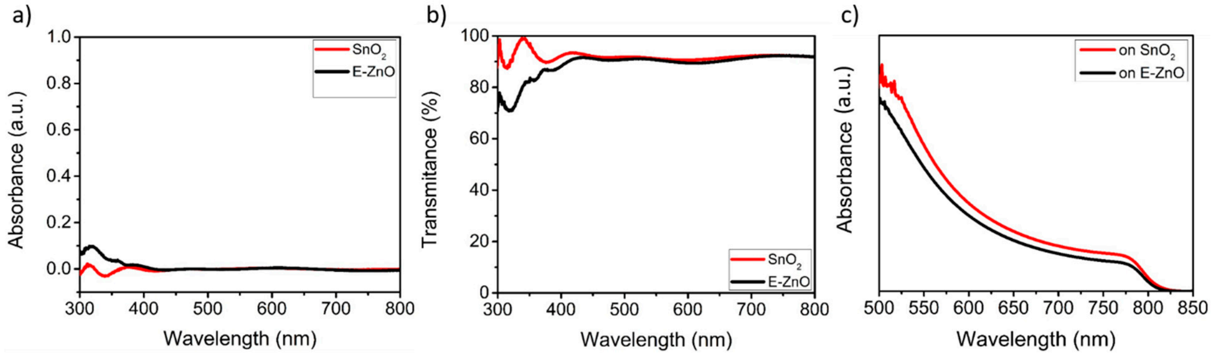

Figure S3. UV-Vis a) Absorption and b) transmittance spectra of the E-ZnO and SnO_2 compact layers deposited on the ITO. c) Optical absorbance of FACsPI on SnO_2 and E-ZnO.

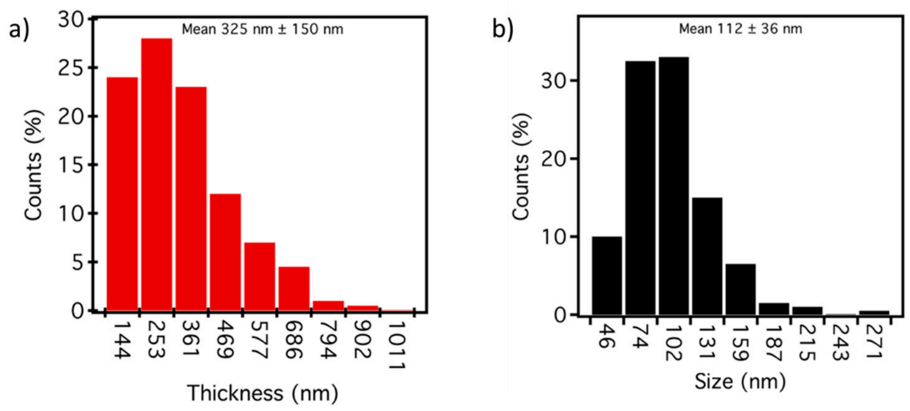

Figure S4. Size distribution of the perovskite grains deposited over a) SnO_2 and b) E-ZnO, mean size ± standard deviation is reported.

Diode model

J/V curves were fitted with a simple diode model represented by the following equations:

$$J = J_{sc} - J_{rec} - J_{sh} \tag{S1}$$

$$J_{rec} = \frac{mK_BT}{qR_{rec0}}\left(e^{\frac{qV_F}{mK_BT}} - 1\right) \tag{S2}$$

$$J_{sh} = \frac{V_F}{R_{sh}} \tag{S3}$$

Where, J_{rec} and J_{sh} are the recombination and shunt currents, respectively, K_B and q are the Boltzmann constant and the electron elementary charge, m, R_{rec0} and R_{sh} are the diode ideality factor, the recombination resistance at V=0 and the shunt resistance, and V_F is the Fermi voltage, obtained by correcting the applied voltage (V_{app}) with the voltage drop due to the series resistance R_s ($V_F = V_{app} - J{*}R_s$).

To obtain a better adjustment, it was considered that according to Equations (S1) to (S3)

$$\frac{dJ}{dV_F} = \frac{-e^{\frac{qV_F}{mK_BT}}}{R_{rec0}} - \frac{1}{R_{sh}} \tag{S4}$$

$$-\frac{dJ}{dV_F} = \frac{1}{R_{rec}} + \frac{1}{R_{sh}} \tag{S5}$$

Where R_{rec} is the recombination resistance as a function of the voltage. Then $\left(-\frac{dJ}{dV_F}\right)^{-1} = R_1$, is the combination in parallel of the recombination and shunt resistances.

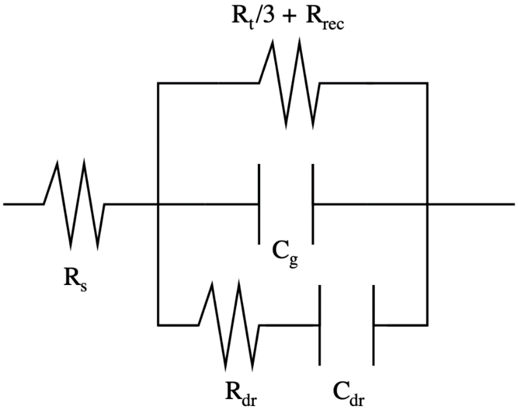

Figure S5. The equivalent circuit used to fit the impedance response of the samples, showing the series resistance R_s, the resistance associated with Transport and recombination processes $R_t/3$ +R_{rec}, the geometrical capacitance C_g, and the relaxation like resistance and capacitance R_{dr} and C_{dr}.[32]

Table S1. Photovoltaic parameters of optimized SnO_2 and E-ZnO based FACsPI perovskite solar cells.

	**PCE (%) (mean ± SD)**	**V_{oc} (V) (mean ± SD)**	**J_{sc} (mA/cm^2) (mean ± SD)**	**FF (%) (mean ± SD)**	**Best PCE (%)**
**SnO_2**	17.06 ± 0.77	1.05 ± 0.01	23.15 ± 0.25	70.1 ± 2.49	17.9
**E-ZnO**	17.16 ± 0.44	1.08 ± 0.01	22.90 ± 0.25	69.5 ± 1.09	18.1

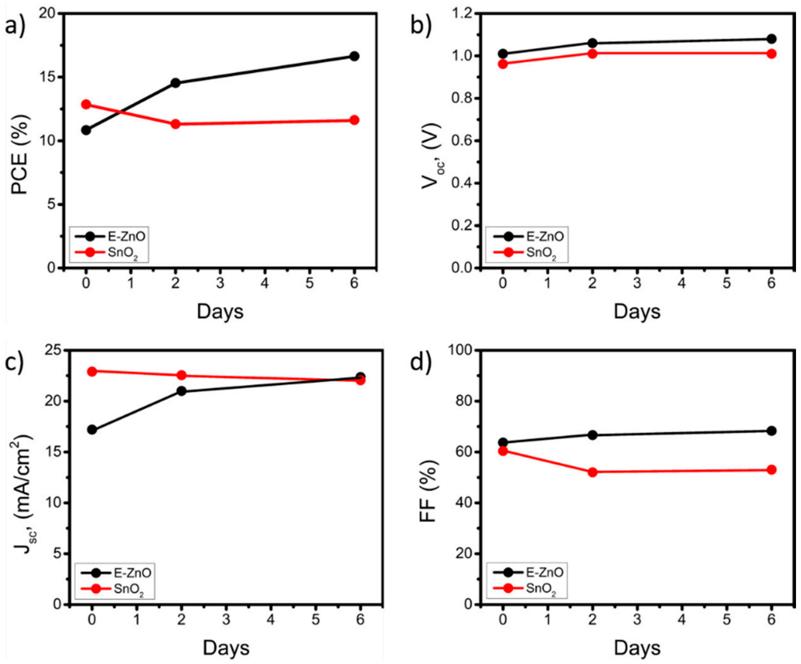

Figure S6. Temporal evolution of photovoltaic parameters the devices using E-ZnO and SnO_2 as ETL; a) PCE, b) V_{oc}, c) J_{sc} and d) FF.

Table S2**. Photovoltaic parameters of SnO_2 and E-ZnO based FACsPI perovskite solar cells during 6 days.**

**Day 0**	**PCE (%) (mean ± SD)**	**V_{oc} (V) (mean ± SD)**	**J_{sc} (mA/cm^2) (mean ± SD)**	**FF (%) (mean ± SD)**
**SnO_2**	12.64 ± 1.27	0.97 ± 0.03	22.84 ± 0.27	57.1 ± 4
**E-ZnO**	10.84 ± 2.51	1.01 ± 0.04	17.04 ± 1.66	62 ± 7
**After 2 days**	**PCE (%) (mean ± SD)**	**V_{oc} (V) (mean ± SD)**	**J_{sc} (mA/cm^2) (mean ± SD)**	**FF (%) (mean ± SD)**
**SnO_2**	11.3 ± 0.48	0.99 ± 0.02	22.3 ± 0.49	51.27 ± 2
**E-ZnO**	14.55 ± 1.45	1.06 ± 0.02	20.48 ± 0.92	66.76 ± 3
**After 6 days**	**PCE (%) (mean ± SD)**	**V_{oc} (V) (mean ± SD)**	**J_{sc} (mA/cm^2) (mean ± SD)**	**FF (%) (mean ± SD)**
SnO_2	11.64 ± 1.94	1 ± 0.02	22 ± 0.99	52.54 ± 6
E-ZnO	15.94 ± 0.95	1.06 ± 0.03	22.09 ± 0.57	68.09 ± 2

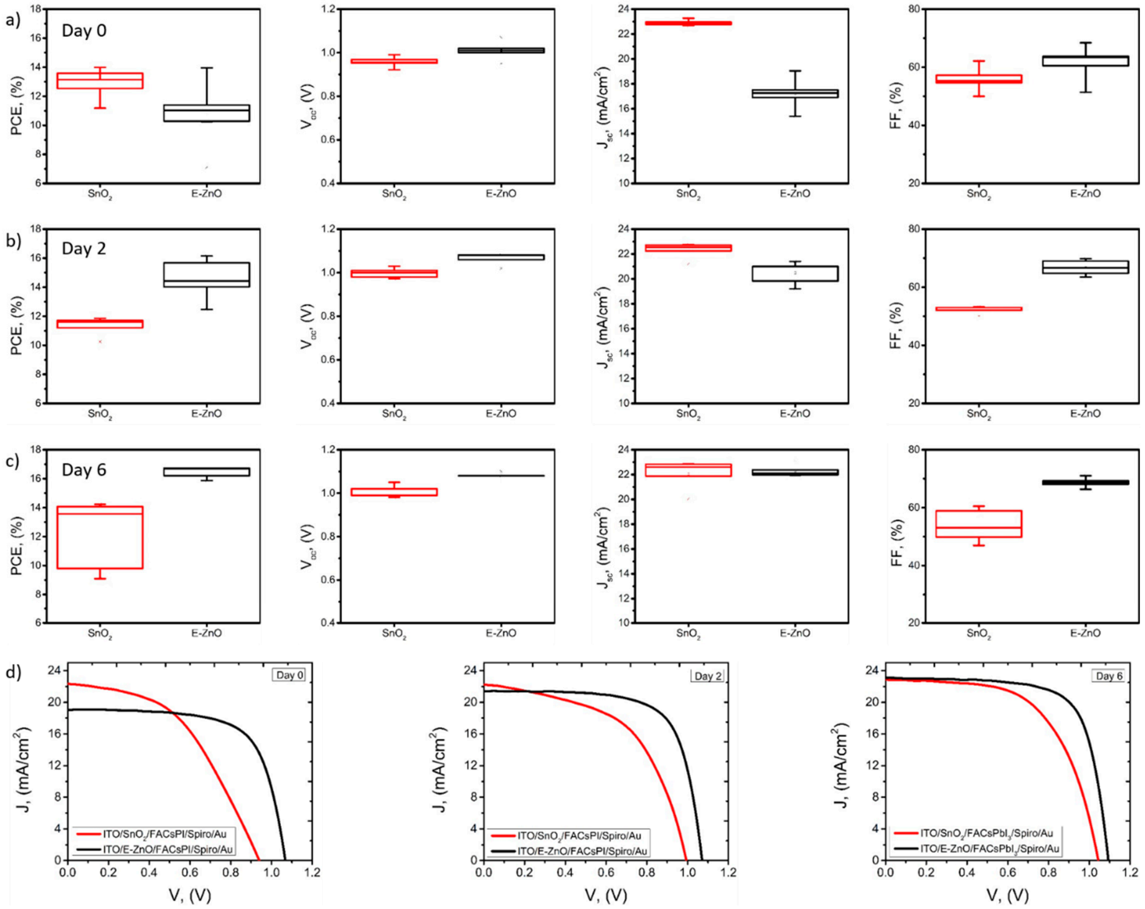

Figure S7. Statistical analysis of photovoltaic parameters of a) day 0, b) day 2, c) day 6 and d) representative J/V curves of the performance evolution of SnO_2 and E-ZnO based FACsPI solar cells.

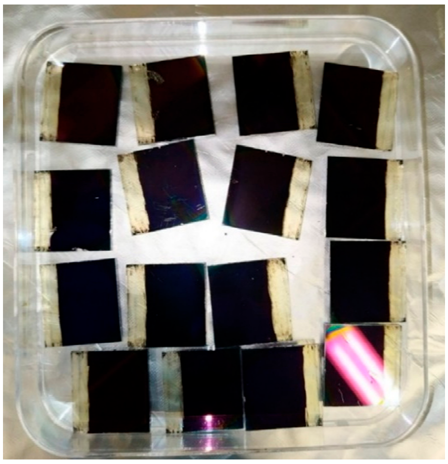

Figure S8. Picture of FACsPI perovskite over SnO_2 and E-ZnO after 2 days in ambient conditions, no degradation is observed.

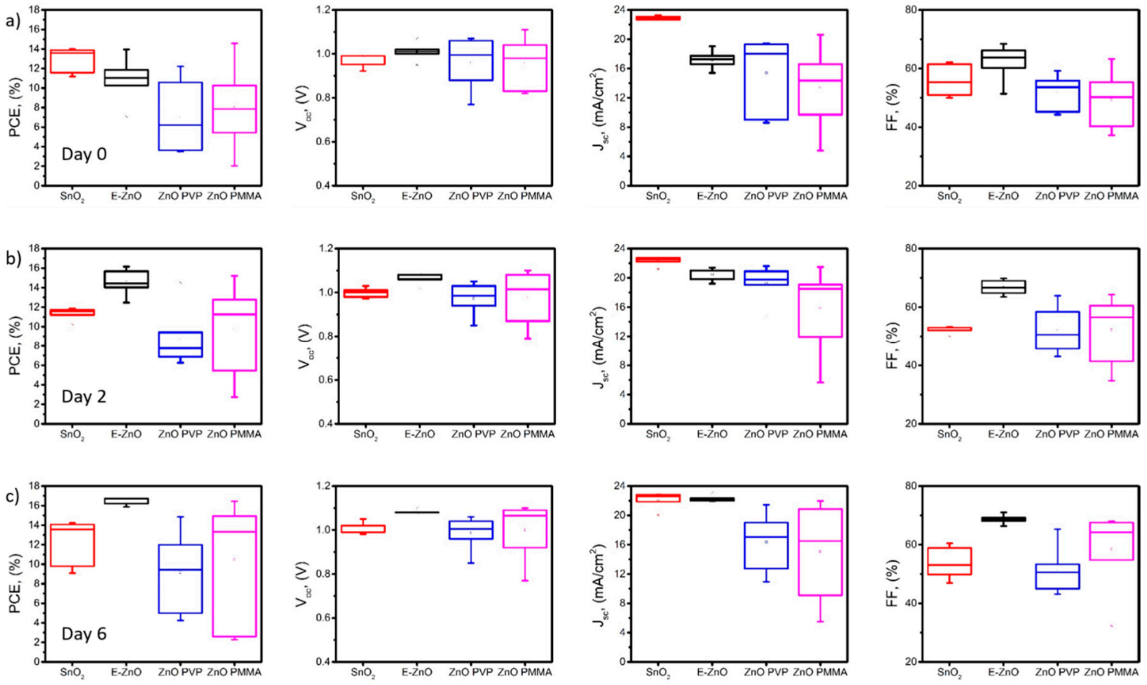

Figure S9. Statistics of photovoltaic parameters of SnO_2, E-ZnO, ZnO/PVP and ZnO/PMMA based FACsPI solar cells at a) day 0, b) day 2 and c) day 6.

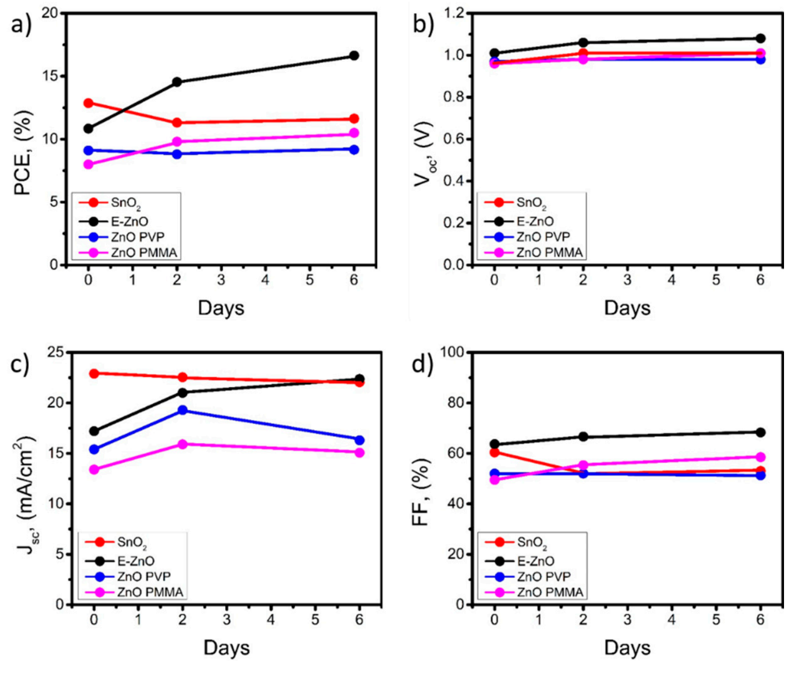

Figure S10. Temporal evolution of a) PCE, b) V_{oc}, c) J_{sc}, and d) FF of FACsPI solar cells with different ETL: SnO_2, E-ZnO, ZnO/PVP, and ZnO/PMMA

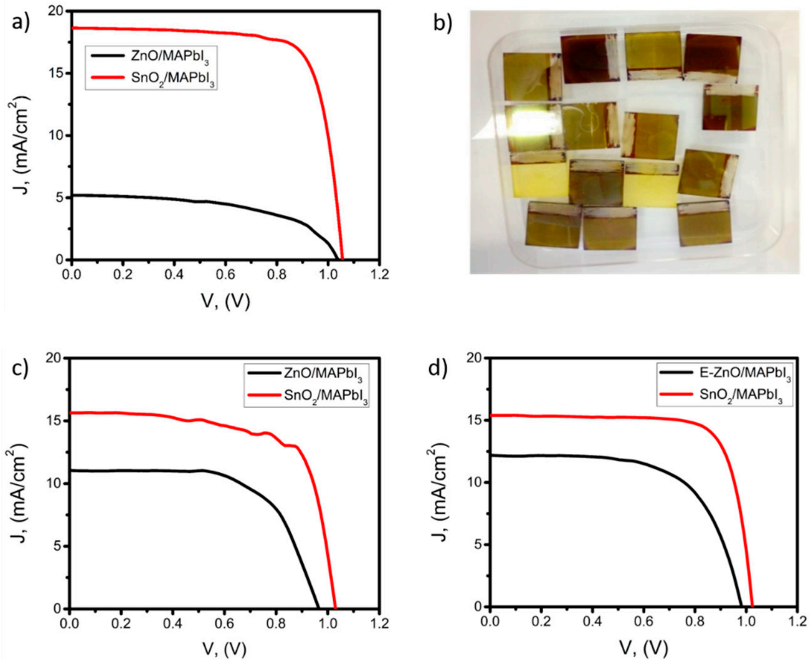

Figure S11. Best J/V curves of ZnO-based $MAPbI_3$ solar cells made with a) synthesis 1; c) synthesis 2 and d) E-ZnO, and b) picture of degraded $MAPbI_3$ perovskite over ZnO layer.

Table S3. Statistic of the $MAPbI_3$ solar cells figures of merits, based on ZnO of synthesis 1, synthesis 2, and E-ZnO.

	PCE (%) (mean ± SD)	V_{oc} (V) (mean ± SD)	J_{sc} (mA/cm^2) (mean ± SD)	FF (%) (mean ± SD)	Best PCE (%)
**FTO/SnO_2/$MAPbI_3$/SPIRO/Au Reference**	12.55 ± 3	1.04 ± 0.03	18.37 ± 0.54	65.27 ± 13	15
**FTO/ZnO/$MAPbI_3$/SPIRO/Au Synthesis 1**	1.67 ± 1	0.76 ± 0.04	4.15 ± 0.73	45.53 ± 12	2.9
**FTO/ZnO/$MAPbI_3$/SPIRO/Au Synthesis 2**	3.5 ± 1.4	0.91 ± 0.01	7.44 ± 1.6	49.25 ± 8.9	6.7
**FTO/E-ZnO/$MAPbI_3$/SPIRO/Au**	6.23 ± 1.3	0.98 ± 0.005	12.09 ± 1.3	52.35 ± 7.3	8.03

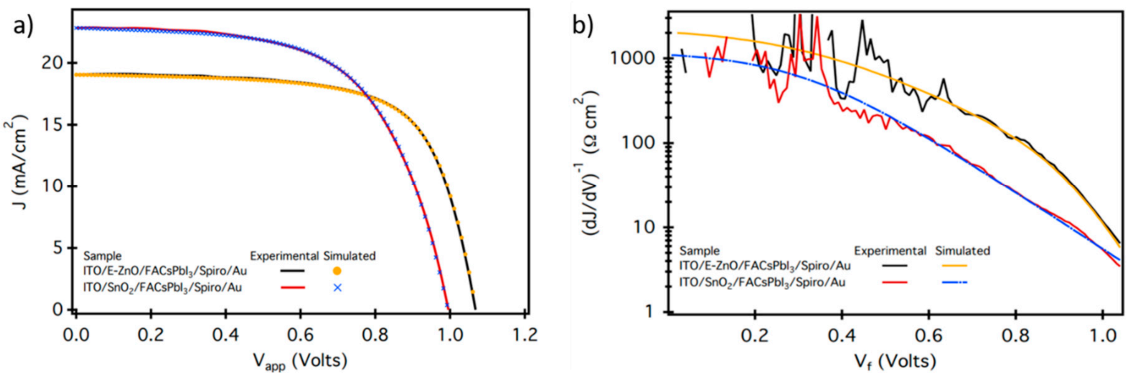

Figure S12. a) Representative fittings of the J/V curves using the diode model compared with the experimental data. All simulated curves had a correlation factor higher than 0.995. b) Representative simulations and experimental data of the resistances in the J/V curves.
